# Supplementary material for: Neuromotor prosthetic to treat stroke-related paresis: N-of-1 trial
Source: Commun Med (Lond). 2022 Apr 7;2:37. doi: 10.1038/s43856-022-00105-8 (PMC9053238; doi:10.1038/s43856-022-00105-8)
Supplement: Supplementary file 1 — Description of Additional Supplementary Files [file 43856_2022_105_MOESM1_ESM.pdf]

Description of Additional Supplementary Files

File Name: Supplementary Data 1.

Description:

Stroke Impact Scale Details. Items with serial improvements are highlighted in green; items showing a decrement are highlighted in pale orange.

File Name: Supplementary Data 2.

Description:

Numerical data used to generate Figures 5, 6, and 7.

File Name: Supplementary Video 1

Description:

MOVIE1_MyoPro_Myoelectric_drop_eraser
11/17/20 25 days post-implant
The participant performs a task using the MyoPro in full myoelectric mode: residual biceps/triceps activity is being used to trigger the elbow motor, and residual wrist flexor activity is being used to trigger the hand motor. (Also note that the participant is wearing a cap and the cables are not connected). This video was recorded 25 days post-implant and demonstrates that residual motor control remains intact less than a month after the four electrode arrays were implanted into neocortex immediately overlying the subcortical stroke. This also provides an example of myoelectric-only performance to which direct neural control may be subsequently compared.

File Name: Supplementary Video 2

Description:

MOVIE2_1D_CURSOR_BCI_CONTROL
46-days post-implant
The participant controls a neural cursor using activity in a 1-dimensional cursor task. This takes place immediately after having built the model in a 1-minute training period where the participant was asked to imagine moving the left hand without moving the left limb in any way. The target cursor is being controlled manually by one of the study investigators. Neural activity comprises

action potentials recorded from the two microelectrode arrays that are more medial and anterior relative to the other pair of arrays, along the precentral gyrus. This video was recorded 46-days after the arrays were implanted.

File Name: Supplementary Video 3

Description:

MOVIE3_1D_CURSOR_BCI_CONTROL_2
46 days post-implant
This is another example where the participant controls a neural cursor using activity in a 1-dimensional cursor task. This takes place immediately after having built the model in a 1-minute training period where the participant was asked to imagine moving the left hand without moving the left limb in any way. The target cursor is being controlled manually by one of the study investigators. Neural activity comprises action potentials recorded from the two microelectrode arrays that are more medial and anterior relative to the other pair of arrays, along the precentral gyrus. This video was recorded 46-days after the arrays were implanted.

File Name: Supplementary Video 4

Description:

The participant is asked to pick up an eraser with the paretic left hand and drop it in a bucket placed at the side of the chair he is sitting in. The eraser is then retrieved and returned to his lap for the task to repeat. The hand motor in this video is being controlled by neural activity recorded from the two microelectrode arrays that are more medial and anterior relative to the other pair of arrays, along the precentral gyrus. Ensemble action potential activity is being processed in real-time through a linear filter model that had previously been built using a one-dimensional cursor task. This video was recorded 48-days after the arrays were implanted.

File Name: Supplementary Video 5

Description:

MOVIE5_DAY53_MYOPRO_BCI_ERASER_DROP
53 days post-implant
This is another example where the participant is asked to pick up an eraser with the paretic left hand and drop it in a bucket placed at the side of the chair he is sitting in. The eraser is then retrieved and returned to his lap for the task to repeat. The hand motor in this video is being controlled by neural activity recorded from the two microelectrode arrays that are more medial and anterior relative to the other pair of arrays, along the precentral gyrus. Ensemble action potential activity is being processed in real-time through a linear filter model that had previously

been built using a one-dimensional cursor task. This video was recorded 53-days after the arrays were implanted.

File Name: Supplementary Video 6

Description: MOVIE6_DAY69_CENTER_OUT_LINEAR
69 days post-implant
The participant controls a neural cursor using activity in a 2-dimensional cursor task. This takes place immediately after having built the model in a 1-minute training period where the participant was asked to imagine moving the left hand without actually moving the left limb in any way. Neural activity comprises action potentials recorded from the two microelectrode arrays that are more medial and anterior relative to the other pair of arrays, along the precentral gyrus. This video was recorded 69-days after the arrays were implanted. The participant was not able to acquire behaviorally useful control of the neural cursor.

File Name: Supplementary Video 7

Description:

MOVIE7_DAY84_MYOPRO_MYO_JEBSEN_PILLBOTTLE
84 days post-implant
The participant is asked to pick up one pill bottle at a time and then move these to a different location and drop them. This is repeated for five pill bottles in a row. Control of the MyoPro powered orthosis is being performed only with residual muscle activity. Residual biceps/triceps activity is being used to trigger the elbow motor, and residual wrist flexor activity is being used to trigger the hand motor. For the hand control, the default is to have the hand open (opposing the participant's spasticity that would cause the hand to close): only when wrist flexor activity exceeds a predetermined threshold will the hand motor close the hand. This video was recorded 84 days after the arrays were implanted.

File Name: Supplementary Video 8

Description:

MOVIE8_DAY84_MYOPRO_BCI_JEBSEN_PILLBOTTLE
84 days post-implant
The participant is asked to pick up one pill bottle at a time and then move these to a different location and drop them. This is repeated for five pill bottles in a row. Residual biceps/triceps activity is being used to trigger the elbow motor of the MyoPro orthosis, and neural activity directly recorded from neocortex is being used to trigger the hand motor. For the hand control, the default is to have the hand open (opposing the participant's spasticity that would cause the hand to close): only when neural population activity exceeds a rolling baseline threshold will the hand motor close the hand. This video was recorded 84 days after the arrays were implanted.

File Name: Supplementary Video 9

Description:

MOVIE9_DAY84_MYOPRO_BCI_BALL
84 days post-implant
The participant is asked to pick up a ball and then drop it into a basket. Residual biceps/triceps activity is being used to trigger the elbow motor of the MyoPro orthosis, and neural activity directly recorded from neocortex is being used to trigger the hand motor. For the hand control, the default is to have the hand open: only when neural population activity exceeds a rolling baseline threshold will the hand motor close the hand; when it drops below this threshold, the hand opens again. This video was recorded 84 days after the arrays were implanted.

File Name: Supplementary Video 10

Description:

MOVIE10_DAY84_MYOPRO_BCI_BALL_2
84 days post-implant
The participant is asked to pick up a ball and then drop it into a basket. Residual biceps/triceps activity is being used to trigger the elbow motor of the MyoPro orthosis, and neural activity directly recorded from neocortex is being used to trigger the hand motor. For the hand control, the default is to have the hand open: only when neural population activity exceeds a rolling baseline threshold will the hand motor close the hand; when it drops below this threshold, the hand opens again. This video was recorded 84 days after the arrays were implanted.

File Name: Supplementary Video 11

Description:

MOVIE11_DAY90_BCI_2Dcursor
90 days post-implant
The participant controls a neural cursor using activity in a 2-dimensional cursor task. This takes place immediately after having built the model in a 1-minute training period where the participant was asked to imagine moving the left hand without actually moving the left limb in any way. Neural activity comprises action potentials recorded from the two microelectrode arrays that are more medial and anterior relative to the other pair of arrays, along the precentral gyrus. This video was recorded 90-days after the arrays were implanted. The participant was not able to acquire behaviorally useful control of the neural cursor.

File Name: Supplementary Video 12

Description:

MOVIE12_MyoPro_Myoelectric_OT_gym
The participant performs a task using the MyoPro in full myoelectric mode: residual biceps/triceps activity is being used to trigger the elbow motor, and residual wrist flexor activity is being used to trigger the hand motor. This video was recorded 14 days after the arrays were surgically removed. The video demonstrates that the ability to use residual muscle activity to perform behaviorally useful tasks remains intact after two neurosurgical procedures (device implantation then device removal) on the cortex overlying a chronic subcortical stroke.
