# Supplementary material for: Neuromotor prosthetic to treat stroke-related paresis: N-of-1 trial
Source: Commun Med (Lond). 2022 Apr 7;2:37. doi: 10.1038/s43856-022-00105-8 (PMC9053238; doi:10.1038/s43856-022-00105-8)
Supplement: Supplementary file 2 — Supplementary Information [file 43856_2022_105_MOESM2_ESM.pdf]

Supplementary Table 1. Clinical Trial Selection Criteria.

Inclusion Criteria
• Must be 18 years or older.<br>• Clinical diagnosis of stroke (hemorrhagic or ischemic, cortical or subcortical), confirmed by brain computer tomography or magnetic resonance imaging, that occurred six or more months prior to enrollment<br>• Must have arm weakness due to stroke.<br>• Participant is willing to comply with all follow-up evaluations at the specified times.<br>• Participant is able to provide informed consent prior to enrollment in the study.<br>• The participant is fluent in English.<br>• > 24 on the Mini Mental Status Examination<br>• Medically stable.<br>• Passive flexion of shoulder in weakened upper extremity with range of > 30 degrees or more<br>• Passive abduction of shoulder in weakened upper extremity with range of > 20 degrees or more<br>• Participant must have a caregiver willing to participate in the study who will help provide care for the surgical site.<br>• Must be willing to live at hospital or at nearby hotel for 90-day duration when implantable components were present.<br>• Plateaued post-stroke recovery with complete or incomplete hemiplegia due to stroke in one upper limb, as measured on two serial occasions (at least one month apart) without improvement, by the following standardized functional assessments of the weaker upper extremity, at their worst:<br>○ Manual Muscle Testing scores of 0/5 (no movement) or 1/5 (palpable contraction in muscle, without movement) in the biceps (elbow flexion), triceps (elbow extension), wrist flexors, wrist extensors or intrinsic hand muscles<br>○ Mild to plegia according to Medical Research Council Scale for Muscle Strength<br>○ Fugl-Meyer Motor Impairment Score of 38 or lower<br>○ Action Research Arm Test (ARAT) score of 35 or lower<br>○ Motricity Index score of 55 or lower<br>• No joint contracture or severe spasticity in affected upper limb precluding the operation of the MyoPro orthotic device.<br>• Sufficient sitting balance to participate with robotic brace activities.<br>• No condition (e.g., severe arthritis, central pain) that would interfere with administration of motor function tests, ability to understand verbal commands and cooperate with test procedures.
Exclusion Criteria
• No medical condition requiring active anti-coagulation with a medication such as heparin, warfarin or rivaroxaban (note that anti-platelet agents such as aspirin or clopidogrel are acceptable)<br>• No active wound healing or skin breakdown issues.<br>• No history of poorly controlled autonomic dysreflexia.<br>• Visual impairment such that extended viewing of a computer monitor would be challenging even with ordinary corrective lenses<br>• Chronic oral or intravenous steroids or immunosuppressive therapy<br>• A score of 23 or lower on Folstein's Mini-Mental Status Examination<br>• Orthopedic conditions of either arm that would affect performance on study

- Untreated psychiatric disturbances that would affect motivation and trial participation
- Medical contraindications for general anesthesia, craniotomy, or surgery.
- Diagnosis of acute myocardial infarction or cardiac arrest within previous 6 months.
- Dementia
- Other implantable devices such as heart/brain pacemakers
- Participants who rely on ventilators
- Co-morbid conditions that would interfere with study activities or response to treatment, which may include:
    - Life expectancy < 3 years
    - Severe chronic pulmonary disease
    - Seizure within three months prior to enrollment
    - History of uncontrolled seizures
    - Local, systemic acute or chronic infectious illness
    - Life threatening cardiac arrhythmias
    - Severe collagen vascular disorder
- Kidney failure or other major organ system failures History of a neurological ablation procedure.
- Labeled contraindication for MRI.
- History of HIV infection or ongoing chronic infection (such as tuberculosis).
- Pregnant or of child-bearing potential and are not taking acceptable methods of contraception.
- Participation in another investigational device or medication trial
- Excessive pain in the paretic upper extremity (> 5 on a 10-point visual analog scale)
- Excessive spasticity at the paretic elbow, wrist, or digits as defined as a score of > 2 on the Modified Ashworth Spasticity Scale
- Participating in any experimental rehabilitation or drug studies
- Moderate to severe apraxia (< 2.5 on the Alexander scale)
- Received phenol injections to any portion of the paretic upper extremity within the past 12 months
- Other conditions or circumstances that, in the opinion of the investigators, would preclude safe and/or effective participation, including severe skin conditions, and/or other sequelae that may be contraindicated for arm orthosis use as well as personal circumstance

Supplementary Figure 1. Cumulative, integrated spike activity and local field potential activity across channels fluctuating with joint position and residual left forearm electromyographic activity. The summed spike activity across channels and run through a leaky integrator [29] appeared to fluctuate with specific residual actions in the left upper extremity, in particular hand movements (blue trace in the third from top panel). Biceps (blue trace), triceps (red trace), wrist flexor (green) and wrist extensor (yellow) trace are shown in the fourth from top panel. The fourth from top panel shows the power spectra from the signal recorded at one arbitrarily selected channel on the same array from where the action potentials shown were recorded; the bottom panel displays the local field potentials as a Z-score computed relative to a 1-minute, eyes-open, at rest (no movement, sitting quietly) pre-trial duration. EMG = electromyography. RMS = root mean square.

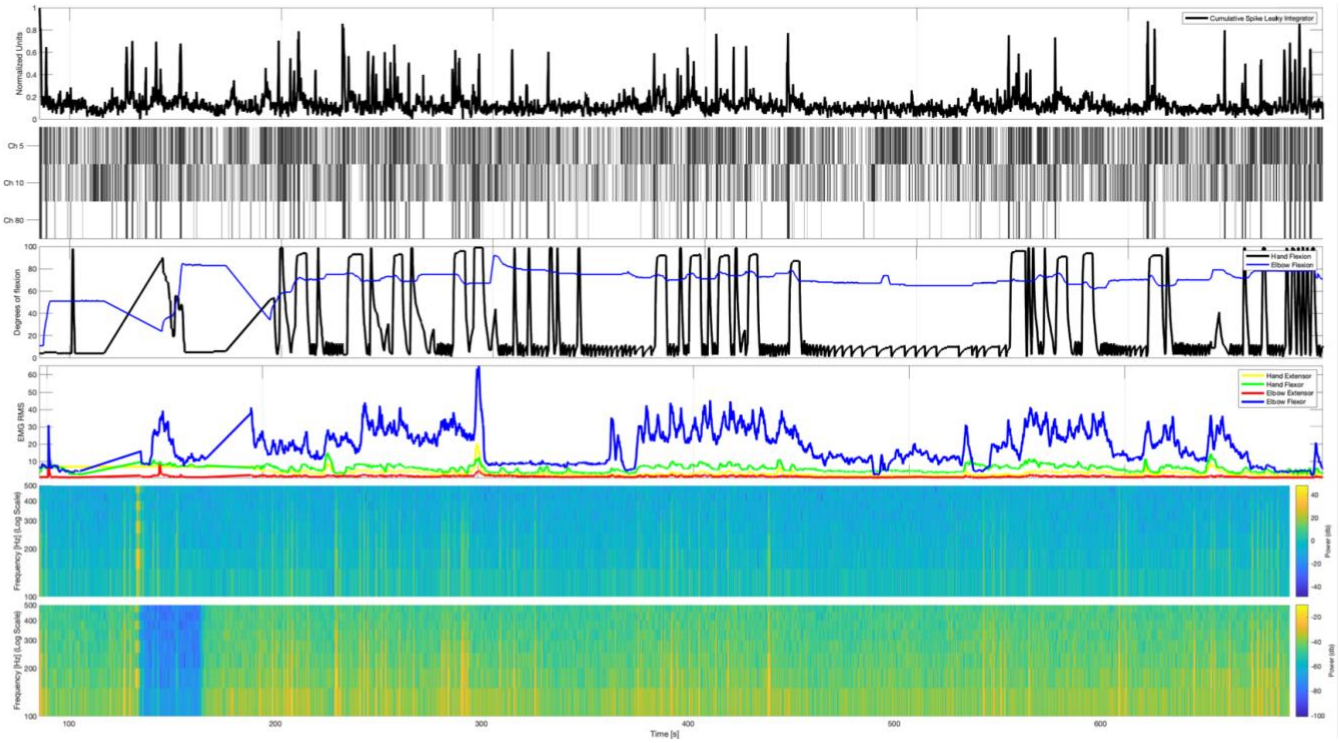

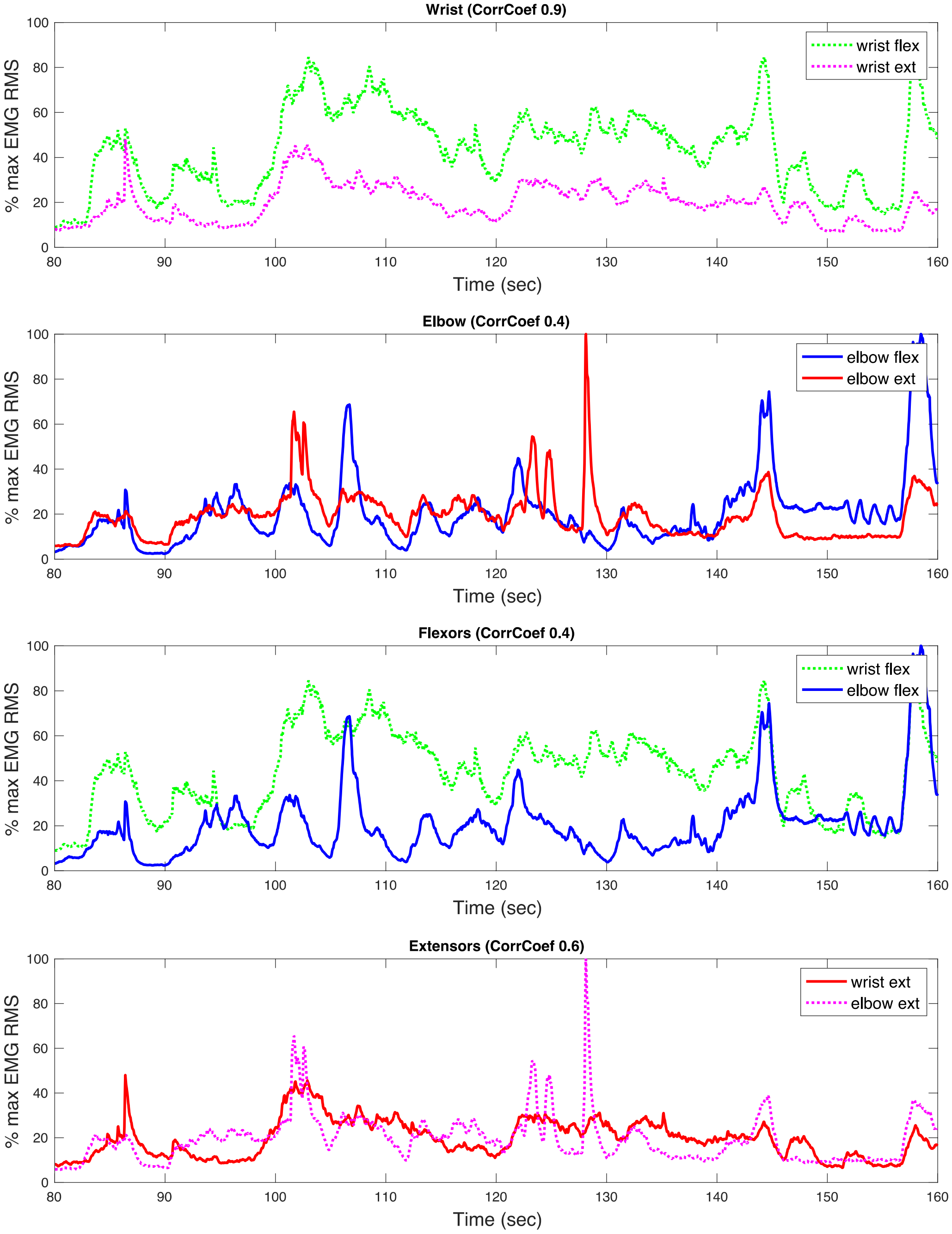

Supplementary Figure 2. Surface electromyographic activity in the paretic arm as the powered brace is being externally controlled by the study team. The MyoPro joint angles are being externally controlled by the trial team, not the participant, who is asked to follow the induced movements to the best of his ability. Note that while the biceps and triceps tend to activate in a reciprocal pattern (expected for normal elbow flexion and extension), the residual wrist extensor activity tracks closely to the wrist flexor activity. For each muscle pair comparison, the correlation coefficient was computed and is shown in the subplot title. This data was recorded on post-implant Day 21. Corr Coef = correlation coefficient. EMG = electromyography. RMS = root mean square. Flex = flexor. Ext = extensor.

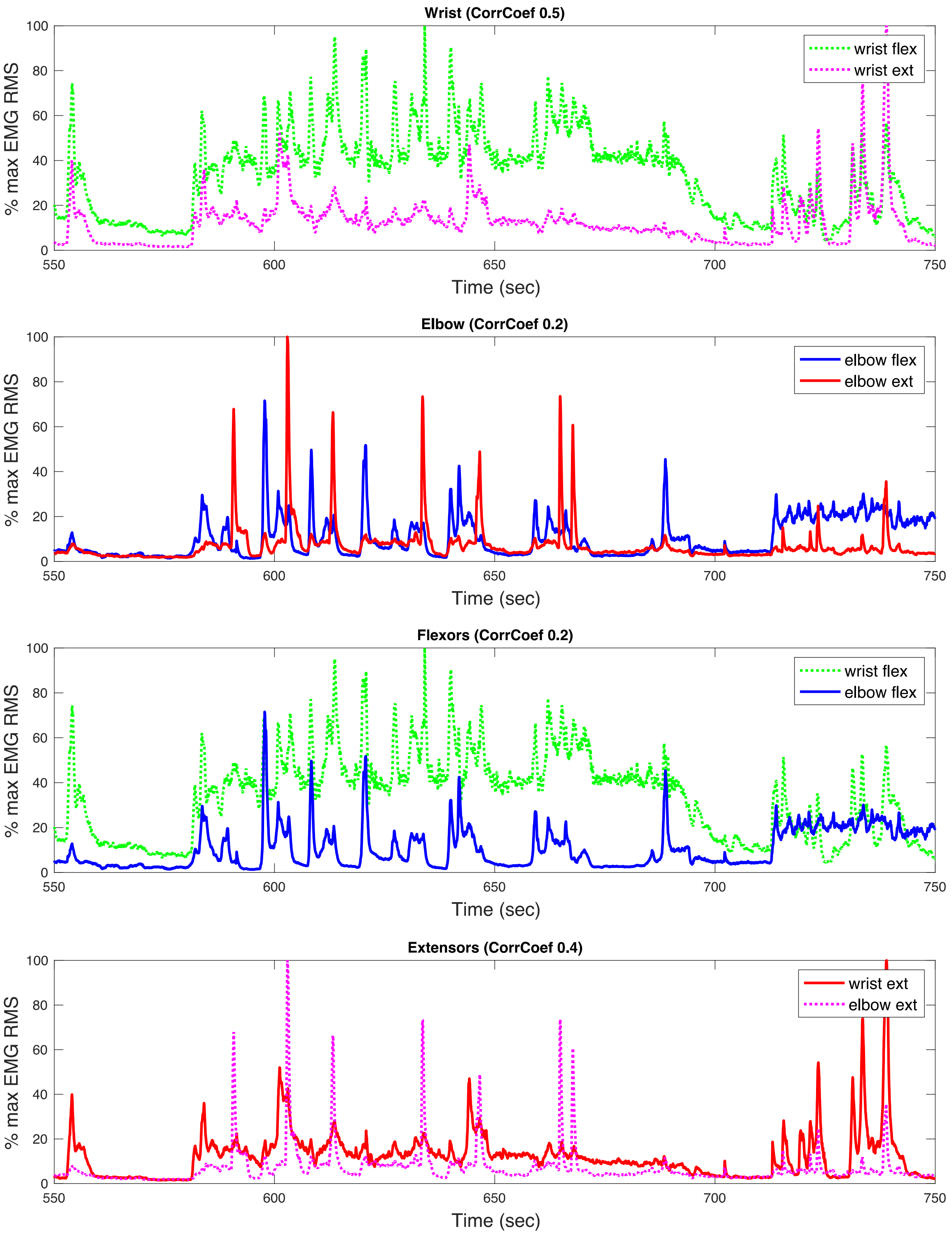

Supplementary Figure 3. Surface electromyographic activity in the paretic limb during voluntary myoelectric control of the powered orthosis. The MyoPro is being controlled by residual muscle activity. This data was recorded on post-implant Day 21. CorrCoef = correlation coefficient. EMG = electromyography. RMS = root mean square. Flex = flexor. Ext = extensor.

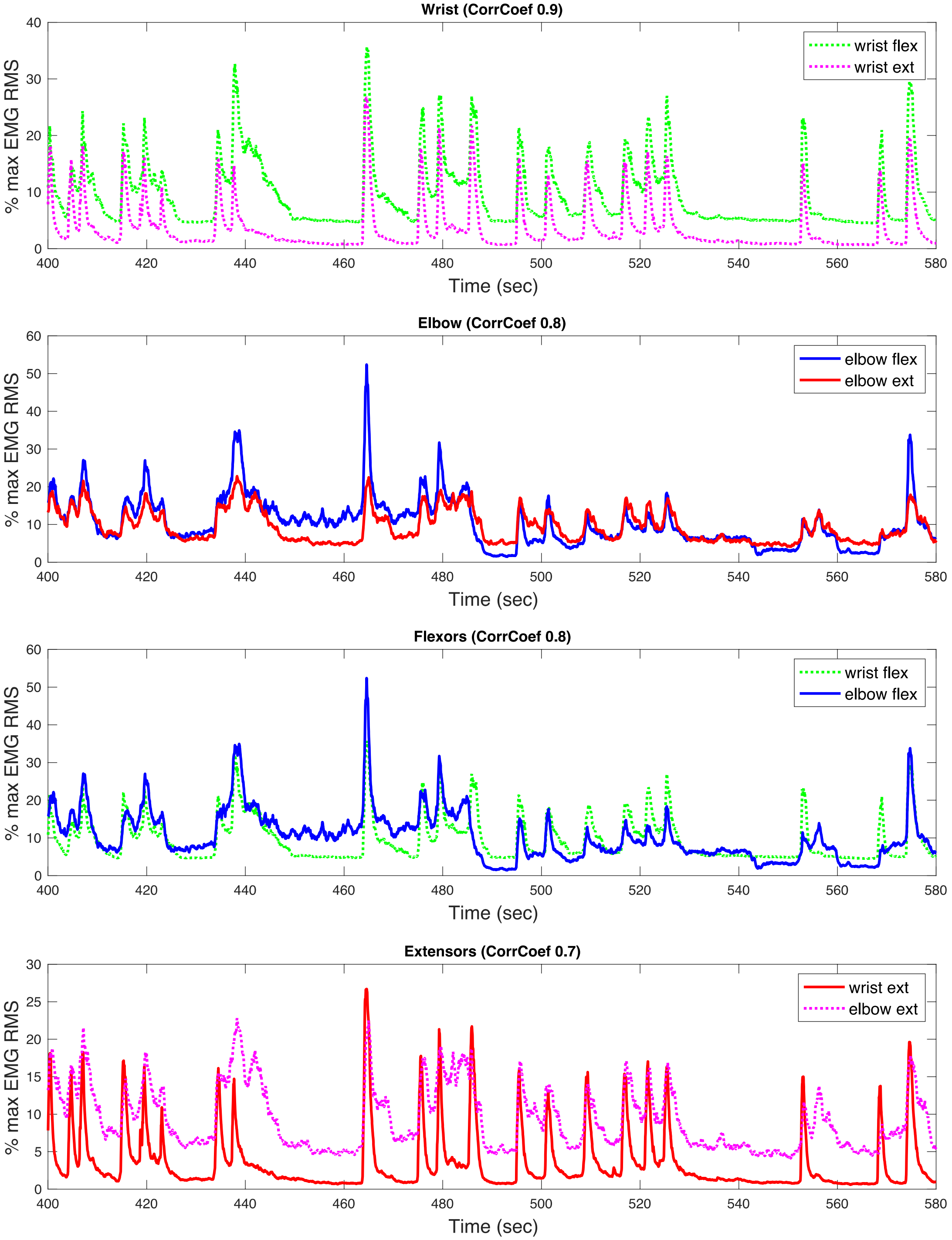

Supplementary Figure 4. Surface electromyographic activity in the paretic limb during voluntary myoelectric control of the powered orthosis. The MyoPro is being controlled by residual muscle activity. This data was recorded on post-implant Day 31. CorrCoef = correlation coefficient. EMG = electromyography. RMS = root mean square. Flex = flexor. Ext = extensor.

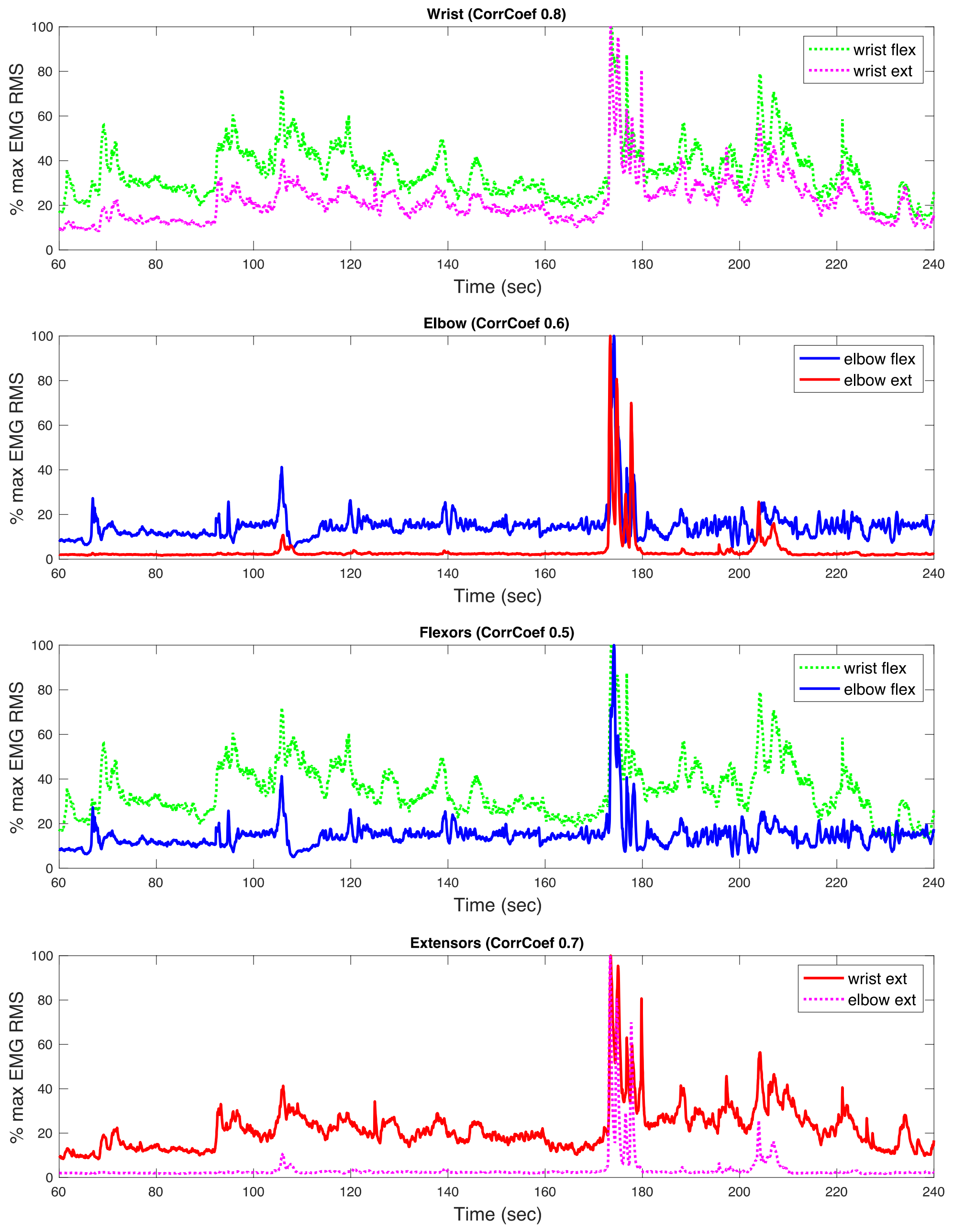

Supplementary Figure 5. Surface electromyographic activity in the paretic limb during voluntary cortical control of the powered orthosis. The MyoPro hand motor is being controlled by the decoded recorded cortical activity (BCI mode) This data was recorded on post-implant Day 83. CorrCoef = correlation coefficient. EMG = electromyography. RMS = root mean square. Flex = flexor. Ext = extensor.

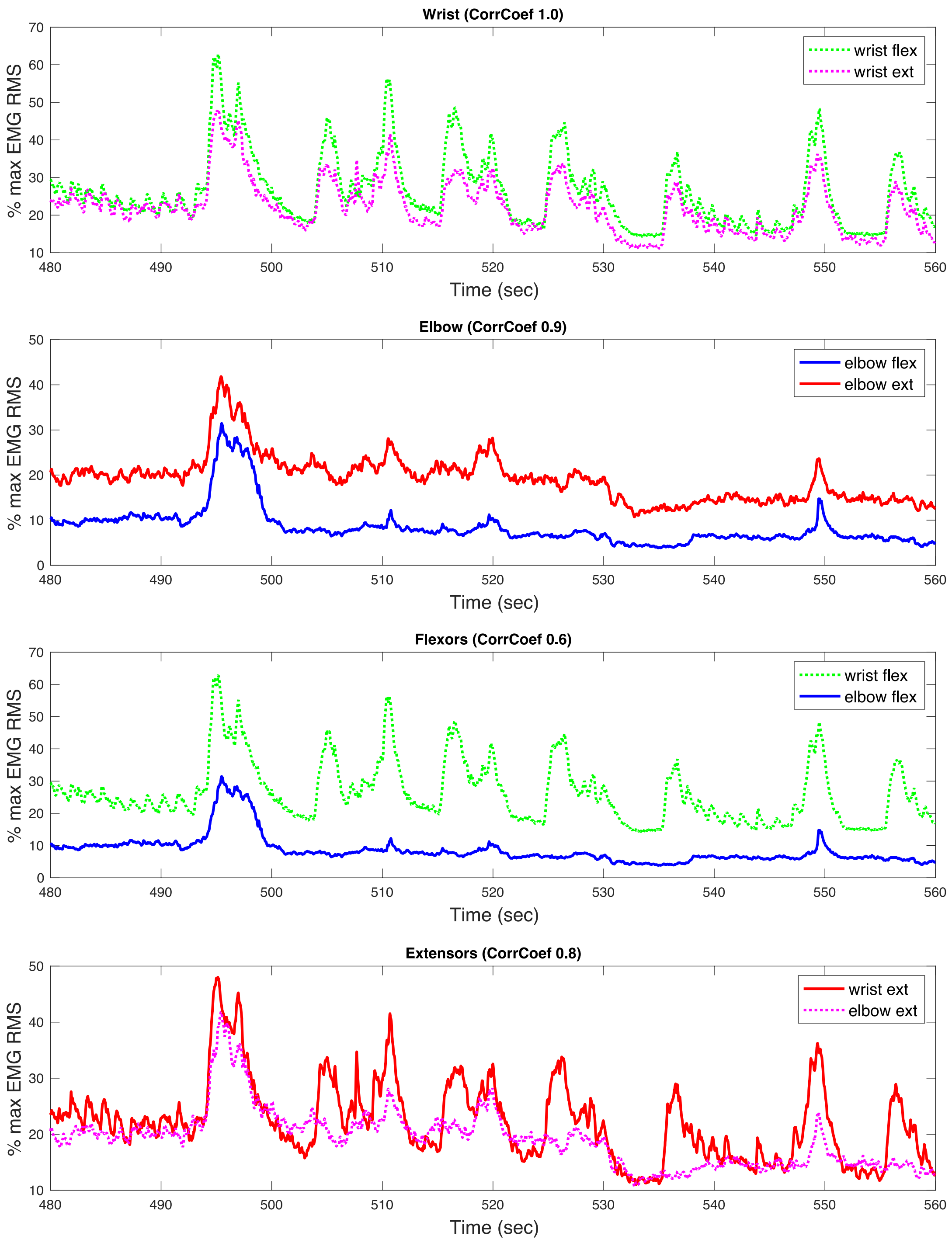

Supplementary Figure 6. Surface electromyographic activity in the paretic limb during voluntary cortical control of the powered orthosis. The MyoPro hand motor is being controlled by the decoded recorded cortical activity (BCI mode) This data was recorded on post-implant Day 84. CorrCoef = correlation coefficient. EMG = electromyography. RMS = root mean square. Flex = flexor. Ext = extensor.

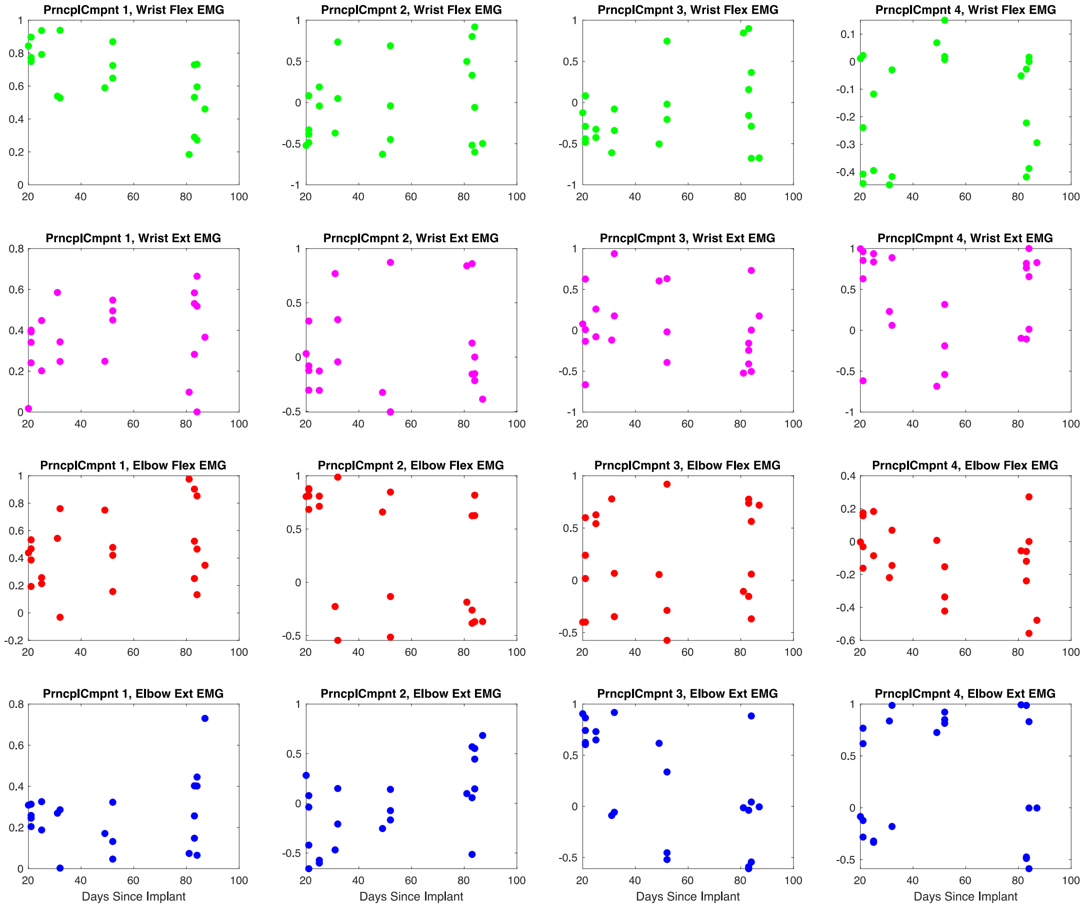

Supplementary Figure 7. Longitudinal trends of principal components derived from surface electromyographic recordings during the time the brain-computer interface was implanted. The coefficient of the principal components derived from the matrix of the four muscle EMG time series (wrist flexors, wrist extensors, biceps, triceps) recorded at different days during the three-month implantation phase of the trial. EMG = electromyography. PrincpCmpnt = principal component. Flex = flexor. Ext = extensor.
