# Supplementary material for: Neuromotor prosthetic to treat stroke-related paresis: N-of-1 trial
Source: Commun Med (Lond). 2022 Apr 7;2:37. doi: 10.1038/s43856-022-00105-8 (PMC9053238; doi:10.1038/s43856-022-00105-8)
Supplement: Supplementary file 18 — Reporting Summary [file 43856_2022_105_MOESM18_ESM.pdf]

nature research

Corresponding author(s): Mijail Serruya

Last updated by author(s): March 1, 2022

Reporting Summary

Nature Research wishes to improve the reproducibility of the work that we publish. This form provides structure for consistency and transparency in reporting. For further information on Nature Research policies, see our Editorial Policies and the Editorial Policy Checklist.

Statistics

For all statistical analyses, confirm that the following items are present in the figure legend, table legend, main text, or Methods section.

n/a	Confirmed	
☐	☒	The exact sample size (*n*) for each experimental group/condition, given as a discrete number and unit of measurement
☒	☐	A statement on whether measurements were taken from distinct samples or whether the same sample was measured repeatedly
☐	☒	The statistical test(s) used AND whether they are one- or two-sided<br>*Only common tests should be described solely by name; describe more complex techniques in the Methods section.*
☒	☐	A description of all covariates tested
☒	☐	A description of any assumptions or corrections, such as tests of normality and adjustment for multiple comparisons
☒	☐	A full description of the statistical parameters including central tendency (e.g. means) or other basic estimates (e.g. regression coefficient) AND variation (e.g. standard deviation) or associated estimates of uncertainty (e.g. confidence intervals)
☒	☐	For null hypothesis testing, the test statistic (e.g. *F*, *t*, *r*) with confidence intervals, effect sizes, degrees of freedom and *P* value noted<br>*Give P values as exact values whenever suitable.*
☒	☐	For Bayesian analysis, information on the choice of priors and Markov chain Monte Carlo settings
☒	☐	For hierarchical and complex designs, identification of the appropriate level for tests and full reporting of outcomes
☒	☐	Estimates of effect sizes (e.g. Cohen's *d*, Pearson's *r*), indicating how they were calculated

Our web collection on statistics for biologists contains articles on many of the points above.

Software and code

Policy information about availability of computer code

Data collection	Software was written in Matlab version 2019b and the Unity 3D engine 2020.1.7.
Data analysis	Analysis was performed in Matlab version 2019b.

For manuscripts utilizing custom algorithms or software that are central to the research but not yet described in published literature, software must be made available to editors and reviewers. We strongly encourage code deposition in a community repository (e.g. GitHub). See the Nature Research guidelines for submitting code & software for further information.

Data

Policy information about availability of data

All manuscripts must include a data availability statement. This statement should provide the following information, where applicable:

- Accession codes, unique identifiers, or web links for publicly available datasets
- A list of figures that have associated raw data
- A description of any restrictions on data availability

Source data for Figures 5, 6 and 7 can be accessed in Supplementary Data 2. The Study Protocol is available at https://doi.org/10.5281/zenodo.6363545 53 and the Analytic Code is available at https://doi.org/10.5281/zenodo.5885015 54. The individual participant data comprises those that underlie the results reported in this article after deidentification (text, tables, figures, appendices, neural and kinematic data). Beginning with publication and ending 5 years following article publication, the data will be made available to researchers who provide a methodologically sound proposal to achieve the aims of the approved proposal. Proposals should be directed to Mijail.Serruya@jefferson.edu . To gain access, data requestors will need to sign a data access agreement.

Field-specific reporting

Please select the one below that is the best fit for your research. If you are not sure, read the appropriate sections before making your selection.

- [x] Life sciences
- [ ] Behavioural & social sciences
- [ ] Ecological, evolutionary & environmental sciences

For a reference copy of the document with all sections, see nature.com/documents/nr-reporting-summary-flat.pdf

Life sciences study design

All studies must disclose on these points even when the disclosure is negative.

Sample size	This is an n-of-1 feasibility study.
Data exclusions	No data was excluded. There were times when the study participant was too tired or unable to continue with a task and in those cases data was not recorded: this has already been cited in the manuscript.
Replication	Reproducibility of the ability to record neural signal time series and correlate them with imagined and attempted movement was sought by repeating recordings and tasks on serial days.
Randomization	Randomization of participants is not applicable in an n-of-1 study.
Blinding	Blinding was not possible: data sets were labeled with the particular task and video analyses could not blind the control mode.

Reporting for specific materials, systems and methods

We require information from authors about some types of materials, experimental systems and methods used in many studies. Here, indicate whether each material, system or method listed is relevant to your study. If you are not sure if a list item applies to your research, read the appropriate section before selecting a response.

Materials & experimental systems

n/a	Involved in the study	
x		Antibodies
x		Eukaryotic cell lines
x		Palaeontology and archaeology
x		Animals and other organisms
	x	Human research participants
	x	Clinical data
x		Dual use research of concern

Methods

n/a	Involved in the study	
x		ChIP-seq
x		Flow cytometry
	x	MRI-based neuroimaging

Human research participants

Policy information about studies involving human research participants

Population characteristics	Not applicable in an n-of-1 study.
Recruitment	Recruitment involved chart review of 1000s of records and then in-person screening of more than 50 participants who met all the selection criteria and were willing to participate.
Ethics oversight	The study was approved by the FDA as an IDE, and by the Thomas Jefferson University IRB. In addition there was a safety monitoring committee that included several faculty who were not on the study.

Note that full information on the approval of the study protocol must also be provided in the manuscript.

Clinical data

Policy information about clinical studies

All manuscripts should comply with the ICMJEguidelines for publication of clinical research and a completedCONSORT checklist must be included with all submissions.

Clinical trial registration	NCT03913286
Study protocol	The full protocol is available at request to the PI.
Data collection	Data was collected from the time of enrollment forward: clinical data was noted on hard copy case report forms at the occupational therapy clinical sites; electrophysiological recording was collected at the temporary housing residence for the participant.

Outcomes	Pre-defined outcomes were developed in coordination with occupational therapy co-investigators on which measures would be ecologically relevant and clinically valid; this was further discussed with the FDA during the IDE application process.

Magnetic resonance imaging

Experimental design

Design type	Block design
Design specifications	Each block comprised 20s rest, 20s active, repeated for 4 blocks
Behavioral performance measures	Compliance assessed by observation from MR control room

Acquisition

Imaging type(s)	Structural, Functional. Diffusion
Field strength	3T
Sequence & imaging parameters	Struct: 3D T2 FLAIR, sagittal, FOV 240x240mm, matrix 240x240 slice 1mm, TR 4800 ms, TE 366.8 ms (eff) , FA 90d; Functional: 2D GRE EPI, axial, FOV 240x240 mm, matrix 80x80, slice 3mm, TR 2000 ms, TE 25 ms, FA 90d; DTI: 2D SE EPI, axial, FOV = 240x240 mm, matrix 96x96, slice 2.5mm, TR 7644, TE 76.8 ms, FA 90d
Area of acquisition	whole brain

Diffusion MRI ☒ Used ☐ Not used

Parameters: 32 directions, b=1000s/mm2, no gating

Preprocessing

Preprocessing software	SPM12, realignment of fMRI, segmentation of structural, coregistration to structural, gaussian smoothing kernel [6x6x6]
Normalization	None, n of 1
Normalization template	NA
Noise and artifact removal	NA
Volume censoring	NA

Statistical modeling & inference

Model type and settings	n of 1, only first level GLM specified
Effect(s) tested	Greater BOLD activation in active as compared with rest

Specify type of analysis: ☒ Whole brain ☐ ROI-based ☐ Both

Statistic type for inference (See Eklund et al. 2016)	voxel wise
Correction	None

Models & analysis

n/a	Involved in the study
☒	☐ Functional and/or effective connectivity
☒	☐ Graph analysis
☒	☐ Multivariate modeling or predictive analysis
